# Gut complement system: a new frontier in microbiota-host communication and intestinal homeostasis

**DOI:** 10.1172/JCI188349

**Published:** 2025-10-01

**Authors:** Xianbin Tian, Lan Zhang, Xinyang Qian, Yangqing Peng, Fengyixin Chen, Sarah Bengtson, Zhiqing Wang, Meng Wu

**Affiliations:** 1 Department of Molecular Microbiology and; 2 Program of Molecular Microbiology and Microbial Pathogenesis, Washington University School of Medicine, St. Louis, Missouri, USA.

## Abstract

The gut microbiota plays a crucial role in maintaining intestinal homeostasis and influencing various aspects of host physiology, including immune function. Recent advances have highlighted the emerging importance of the complement system, particularly the C3 protein, as a key player in microbiota-host interactions. Traditionally known for its role in innate immunity, the complement system is now recognized for its interactions with microbial communities within the gut, where it promotes immune tolerance and protects against enteric infections. This Review explores the gut complement system as a possibly novel frontier in microbiota-host communication and examines its role in shaping microbial diversity, modulating inflammatory responses, and contributing to intestinal health. We discuss the dynamic interplay between microbiota-derived signals and complement activation, with a focus on the C3 protein and its effect on both the gut microbiome and host immune responses. Furthermore, we highlight the therapeutic potential of targeting complement pathways to restore microbial balance and treat diseases such as inflammatory bowel disease and colorectal cancer. By elucidating the functions of the gut complement system, we offer insights into its potential as a target for microbiota-based interventions aimed at restoring intestinal homeostasis and preventing disease.

## Introduction

The gastrointestinal tract is the body’s most immunologically active and microbially dense environment, and it demands finely tuned mechanisms to maintain homeostasis and defend against pathogens. The complement system, which has been classically recognized as a sentinel of the innate immune response, is increasingly being appreciated for its nuanced roles within the local environment. Complement components are not confined to systemic circulation but can be locally produced and activated at mucosal sites, including the gastrointestinal tract. Within this unique niche, the gut complement system orchestrates a delicate balance among microbial surveillance, barrier integrity, and immune modulation, positioning it as a critical yet underexplored mediator of microbiota-host crosstalk. In this Review, we discuss recent advances in our understanding of local complement production with a focus on the gut mucosa, while drawing comparisons to complement activity at other mucosal sites.

## Local biosynthesis and unique adaptations of intestinal complement

The systemic complement system comprises over fifty soluble proteins, most of which are synthesized by hepatocytes in the liver (1, 2). However, extrahepatic expression and synthesis of complement system proteins have been demonstrated, particularly in in vitro cultures of cells derived from humans and guinea pigs (3–6). Initial evidence for local complement synthesis in the gut emerged from studies showing that small intestinal epithelial cells (IECs) from guinea pigs could synthesize hemolytically active C1 (7, 8). This finding was later verified in isolated segments of human colon and ileum, where locally produced C1 was detected through incorporation of radiolabeled amino acids (9). More recently, intestinal macrophages have been identified as the primary source of C1q in the gut (10).

Among complement components, C3 has received particular attention for its role in the intestine because of its central role in the complement system. C3 production has been detected as early as 11 weeks of gestation in human fetal colon cultures (11) as well as in biopsies from both the small intestine and colon of adult (12). Subsequent studies have established that IECs are capable of producing C3. Human colon carcinoma cell lines (Caco-2, T84, HT-29) were shown to synthesize C3 in vitro, in contrast to the embryonic intestinal cell line INT407, which lacked this capacity (13, 14). C3 expression has also been reported in murine IEC lines and primary epithelial cells isolated from ileal and colonic tissues, with expression levels upregulated by LPS stimulation (15). In mouse models of dextran sodium sulfate–induced (DSS-induced) colitis, C3 expression and protein production by IECs are markedly increased (15).

These observations have largely been limited to in vitro, ex vivo, or inflammatory models, and the cellular sources of C3 under homeostatic conditions in the gut remained poorly defined. Our recent work, utilizing flow cytometry and single-cell RNA sequencing (scRNA-Seq) in C3-tdTomato reporter mice, demonstrated that epithelial cells, myeloid populations, and stromal cells all express C3 in the colon during homeostasis (16). Using in situ RNAscope and immunofluorescence, we localized C3-expressing stromal cells within isolated lymphoid follicles. Moreover, in vitro stimulation of colonic stromal cells with microbe-derived products — including formalin-fixed or heat-killed bacteria, as well as defined microbe-associated molecular patterns (MAMPs) such as LPS, peptidoglycan, lipoteichoic acid, and the synthetic triacylated lipopeptide Pam3CysSerLys4 (Pam3CSK4) — was sufficient to induce C3 production.

In addition to C3, IEC lines have been shown to produce other early complement components such as C4 and factor B (13, 14). Strikingly, however, these cells do not produce the terminal complement components (C5–C9) required for membrane attack complex (MAC) formation (14). Consistently, epithelial, stromal, and CD45^+^ immune cells in the intestine did not express appreciable levels of C5–C9 transcripts, and C5 protein remains undetectable in the gut lumen, even during infection, while C3 is robustly upregulated and secreted. Human intestinal scRNA-Seq data mirror these findings, showing that C1q (composed of C1qa, C1qb, C1qc trimers) is predominantly expressed by macrophages, whereas C3 is prominently detected in stromal cells, and C5–C9 are not highly expressed (17). These findings are consistent with a study in a human cohort with small intestinal bacterial overgrowth (SIBO), where C3 deposition was observed without evidence of terminal complement complex (C5–C9) activation (18).

Complement proteins, particularly C3, have also been detected directly in the intestinal lumen. C3 was identified in luminal aspirates from patients with small intestinal infections (18) and in jejunal fluids from both patients with Crohn’s disease (CD) and individuals acting as healthy controls (19). Recent gnotobiotic mouse studies further demonstrated that luminal C3 is present in the feces of both healthy mice and humans, with its levels modulated by the gut microbiota (16). Germ-free and antibiotic-treated mice show reduced levels of fecal C3, whereas conventionally raised mice with certain microbiota communities show increased levels of fecal C3. However, the precise microbial signals governing this regulation remain unclear. While several MAMPs have been identified as inducers of C3 in cultured epithelial and stromal cells, the specific microbial molecules and host-sensing mechanisms, particularly in stromal cells, are yet to be elucidated.

Functionally, C3b deposition on mucosa-associated bacteria has been demonstrated during DSS-induced colitis (15), raising questions about the role of complement in host-commensal interactions. Notably, C3-deficient mice exhibit no major shifts in gut microbiota composition at baseline (16), suggesting that, while C3 can opsonize commensals, it does not drive their elimination under homeostatic conditions. This is likely due to the absence of MAC-mediated lysis and the limited presence of phagocytes within the lumen in steady state. In contrast, during infection, phagocytes such as neutrophils and macrophages are recruited to the gut mucosa to clear pathogens like *Citrobacter rodentium* and enterohemorrhagic *E. coli* (16).

Interestingly, complement-mediated defense in the gut can also be influenced by other sources. For example, Xu et al. demonstrated that complement proteins in maternal milk can suppress Gram-positive bacteria like *Staphylococcus lentus* in the neonatal gut in a C1-, C3-, and MAC-dependent manner (20). This indicates that a unique protective mechanism operates during early life. Conversely, in adult intestines, C1q — although abundant — appears dispensable for defense against luminal pathogens (10, 16). Instead, intestinal C1q, produced by muscularis macrophages, has been implicated in regulating gut motility through modulation of neuronal gene expression (10), echoing its established role in synaptic pruning in the brain (21, 22). This suggests that gut-resident C1q may serve neuromodulatory functions distinct from its canonical role in immunity at the adult stage. Table 1 summarizes known sources, locations, and functions of complement components identified in the intestine.

**Figure 1:**
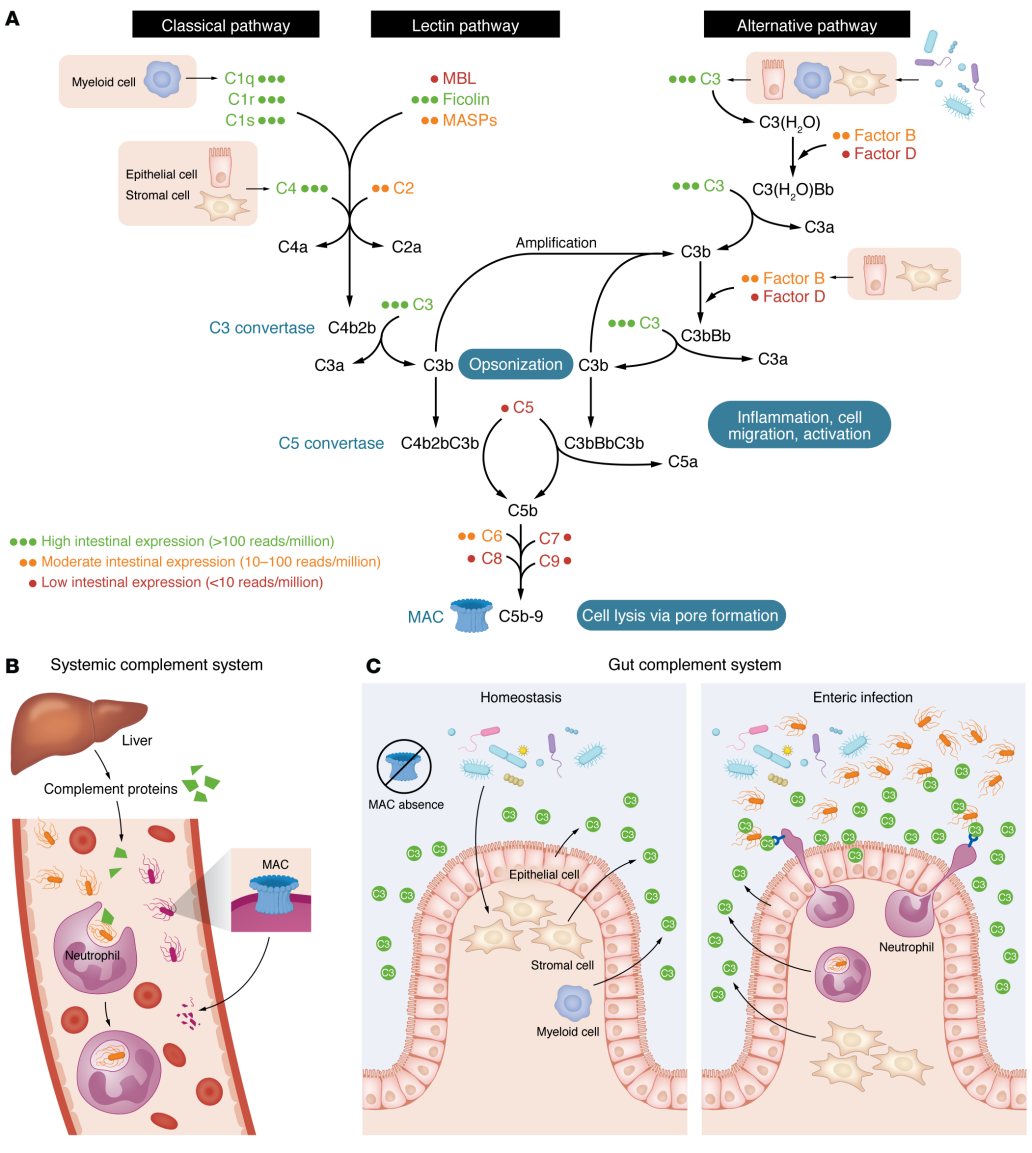
Unique adaptations of the intestinal complement system compared with the systemic complement system. (**A**) Gut-specific complement expression profile. Complement components involved in the three major activation pathways — classical, lectin, and alternative pathways — are depicted, each converging on C3 activation and leading to effector functions, including opsonization, inflammation, and membrane attack complex (MAC) formation, as in the systemic complement system. However, the intestinal complement system exhibits a locally adapted, restricted expression profile. Indicated expression levels reflect transcriptomic data from the intestine under homeostatic conditions (16): green (three dots) indicates high expression (>100 reads/million), orange (two dots) denotes moderate expression (10–100 reads/million), and red (one dot) represents low expression (<10 reads/million). Intestinal cell types responsible for producing these components are shown in pink rectangles (e.g., stromal cells, myeloid cells, and epithelial cells). (**B**) Systemic complement function. The liver is the primary source of circulating complement proteins. In the sterile environment of the bloodstream, these proteins support immune defense through opsonization, recruitment of immune cells, phagocytosis, and direct pathogen killing via MAC-mediated lysis. (**C**) Gut complement function during homeostasis and infection. Under homeostatic conditions (left), C3 is primarily expressed by stromal, myeloid, and epithelial cells in the intestine. Due to limited phagocyte presence and the absence of MAC, this locally produced C3 does not eliminate commensal microbiota. During enteric infection (right), pathogen exposure enhances complement activation, C3 deposition, and promotes neutrophil recruitment and phagocytosis, thereby supporting pathogen clearance.

Collectively, the intestinal complement system displays unique adaptations (Figure 1). It lacks terminal components required for MAC formation, suggesting that bacterial killing relies on phagocytosis rather than direct lysis. This adaptation likely reflects the need for immune tolerance in a microbiota-rich environment, contrasting with the sterile bloodstream where complement-mediated lysis is critical. The alternative pathway is the principal complement activation route in the gut, supporting amplification capacity without compromising microbial tolerance. Intriguingly, the complement profile in the gut resembles that of ancient cnidarians, which possess C3 and the activation proteases factor B and MASP (23). This evolutionary parallel suggests that the gut’s complement system represents an ancient, nonlytic defense mechanism tailored to the mucosal environment.

These distinct features underscore the evolutionary balance the host has achieved between immune defense and tolerance. Continued investigation is essential to unravel the precise molecular pathways by which the gut complement system interfaces with the microbiota, maintains homeostasis, and contributes to disease pathogenesis.

## Complement receptor expression in the intestine

Complement receptors are typically categorized into two primary groups: anaphylatoxin receptors and opsonin receptors. Anaphylatoxin receptors include C3a receptor (C3aR), C5a receptor 1 (C5aR1/CD88), and C5a receptor 2 (C5aR2), while opsonin receptors include CR1 (CD35), CR2 (CD21), CR3 (CD11b/CD18), CR4 (CD11c/CD18), and CRIg (complement receptor of the immunoglobulin superfamily, also known as V-set and immunoglobulin domain-containing 4 [VSIG4]) (24). Table 2 summarizes complement receptor expression in the gut, specifying cell types and known functions. Using floxed fluorescent reporter knock-in mouse models, the group led by Jörg Köhl mapped the expression of the anaphylatoxin receptors C3aR, C5aR1, and C5aR2 in various tissues, including the small intestinal lamina propria (LP) (25–27). They found that C3aR is prominently expressed in the small intestinal LP, with high levels observed on macrophages, CD103^+^/CD11b^+^ conventional DCs, and plasmacytoid DCs, while lower expression is found in subsets of type 3 innate lymphoid cells (ILC3s) and epithelial cells (27). Notably, neutrophils do not express C3aR under steady-state conditions, but its expression is upregulated following LPS stimulation (28). Interestingly, in LP macrophages, C3aR is predominantly localized intracellularly (27), suggesting the involvement of intracellular complement activity, or “complosome,” in regulating intestinal inflammation and antigen presentation (29). Furthermore, C3aR has been detected at both mRNA and protein levels in mouse small intestinal organoids (enteroids) and IEC lines such as T84 and Caco-2 (30). In these models, LPS-induced C3a production triggers proinflammatory signaling through C3aR (15). Additionally, C3aR is expressed on Lgr5^+^ intestinal stem cells within crypt organoids, where it plays a role in promoting stem cell expansion (31).

In contrast to the widespread expression of C3aR in the LP, C5aR1 expression is more restricted, appearing predominantly on LP macrophages in reporter mice (26). C5aR2, by contrast, shows a broader distribution, being expressed on macrophages, DCs, and eosinophils (25). Functionally, C5aR1 is a canonical GPCR that mediates strong proinflammatory responses upon C5a binding, whereas C5aR2 is an atypical GPCR with a context-dependent role, exhibiting both anti- and proinflammatory functions (32). Using a combination of immunofluorescence, flow cytometry, and scRNA-Seq, C5aR1 expression was identified on lysozyme-expressing DCs, where it triggers signaling pathways that promote antigen cross-presentation and CD8^+^ T cell immunity (33). Notably, C5a receptors are not expressed in primary IECs or IEC-1 cell lines at either the mRNA or protein level (15, 34, 35), consistent with the absence of luminal C5. However, C5aR1 and C5aR2 expression has been reported in IEC lines such as T84, HT-29, and Caco-2 (36). Additionally, C5aR1 has been shown to localize to the apical surface of human M-like cells (differentiated Caco-2 cells cocultured with Raji cells) and mouse Peyer’s patch M cells, where it facilitates mucosal antigen uptake (37).

The opsonin receptors are broadly expressed on immune cells, including neutrophils (CR1, CR3, CR4), macrophages (CR1, CR3, CR4, CRIg), DCs (CR1, CR3, CR4, CRIg), B cells (CR1, CR2, CR4), and T cells (CR1) (24). However, the question of whether tissue-resident immune cells, such as intestinal macrophages, exhibit distinct complement receptor profiles shaped by their local microenvironment remains unresolved. CRIg is best characterized by its high expression on liver-resident macrophages, particularly Kupffer cells (38). However, accumulating evidence demonstrates that CRIg is also expressed on tissue-resident macrophages (TRMs) in the small intestine and colon (39). Importantly, CRIg expression in TRMs is postnatally regulated by microbial signals. Its expression is markedly upregulated around the time of weaning but remains low during the neonatal period, suggesting that gut microbiota colonization is a key driver of CRIg induction. This microbiota-dependent regulation is further supported by findings showing a significant reduction in both the frequency of CRIg^+^ TRMs and the per-cell expression levels of CRIg in the colons of mice treated with broad-spectrum antibiotics to deplete the microbiota (39). Functionally, these colonic CRIg^+^ macrophages have been shown to mediate clearance of microbiota-derived extracellular vesicles, implicating them in the maintenance of intestinal homeostasis (40). CR3 has been occasionally detected in the small intestinal submucosa (41), and immunohistochemical studies have revealed CD11b expression on polymorphonuclear leukocytes, CD11c on T lymphocytes, and CD68^+^ macrophages (42). Intriguingly, crypt epithelial cells have also been reported to express CD11b and CD11c, suggesting potential roles for these receptors beyond traditional immune cell contexts (42).

## Complement regulatory proteins in the gut

The expression patterns of complement regulatory proteins (CRPs), including CD55 (decay-accelerating factor [DAF]), CD46 (membrane cofactor protein [MCP]), and CD59 (protectin), have been extensively characterized in both normal and diseased gastrointestinal tissues through immunohistochemical analysis of human tissue samples (43–45). Table 3 summarizes the cellular source, localization, and functions associated with CRPs characterized in the gut.

Under normal conditions, CD55 exhibits restricted apical expression in the small intestinal crypt epithelium but is absent from other regions of the small intestine as well as from the gastric and colonic segments in these samples. However, CD55 expression is significantly upregulated in inflamed tissues, such as those in CD, ulcerative colitis (UC), and celiac disease (43). Deficiency in CD55 is associated with CHAPLE disease, a condition characterized by complement hyperactivation, angiopathic thrombosis, and protein-losing enteropathy, often presenting as primary intestinal lymphangiectasia (46). While CD55 serves a protective role in preventing complement-mediated damage during inflammation, its inhibitory function can be exploited in cancer. Elevated expression of CD55 has been observed in colon cancer tissues compared with adjacent normal tissues, as shown by tissue microarray analyses (44), with similar upregulation noted in colorectal adenomas and carcinomas (47). The human colon carcinoma cell lines Caco-2, T84, and HT-29 also express CD55, providing models for studying its regulation (14). In vitro studies have demonstrated that the induction of CD55 in IECs is strongly driven by IL-4 and TNF-α at both the protein and mRNA levels, moderately by IL-1β, and unaffected by IL-2, IL-6, IL-8, IL-10, or IFN-γ (48–51). Moreover, TNF-α treatment delays CD55 mRNA degradation, while IL-4 does not, suggesting that IL-4 and TNF-α induce CD55 mRNA at the transcriptional and posttranscriptional levels, respectively (48, 49). Additionally, the soluble form of CD55 is detected in the culture supernatant of the HT-29 cell line, providing a theoretical basis for the correlation between fecal CD55 levels and UC severity (52). The protective mucosal protein intestinal trefoil factor has also been shown to enhance CD55 expression through NF-κB activation and mRNA stabilization during mucosal injury (53).

CD59 exhibits weak apical expression in gastric pit and small intestinal villus/crypt epithelia but is strongly expressed in the normal colon. During inflammation, CD59 expression increases in the stomach and small intestine but remains unchanged or reduced in the colons of patients with UC and CD (43, 54). In contrast, CD59 is significantly upregulated in colorectal cancer (CRC) tissues, potentially facilitating tumor evasion of complement-mediated lysis (44, 45, 55). Flow cytometry analysis has further revealed that TNF-α, IL-1β, and IFN-γ enhance CD59 expression in HT-29 cells in a dose-dependent manner (55).

CD46 is constitutively expressed basolaterally across the gut epithelium, with stable expression levels during inflammation (43), although it shows notable upregulation in colon cancer (44, 45). Unlike CD55 and CD59, CD46 expression in HT-29 cells is selectively induced by IFN-γ, rather than by TNF-α or IL-1β (51). Beyond its role in complement regulation, CD46 modulates epithelial barrier dynamics by interacting with the Ste20/SPS1-related kinase (SPAK) and E-cadherin, disrupting epithelial tight junctions, increasing paracellular permeability to pathogens like *E*. *coli*, and simultaneously promoting epithelial cell proliferation and repair (56). However, these findings, which suggest that CD46 plays a central role in both compromising barrier integrity and enhancing tissue repair, are derived from in vitro models and, thus, require further validation in vitro.

Current knowledge regarding CRPs in the gut relies largely on human biopsies and cell lines. To better understand their functional roles and causal relationships, advanced models such as organoids and animal studies are needed. Collectively, the distinct spatial and disease-specific expression patterns of CD55, CD59, and CD46 underscore their specialized roles in gut immunity and pathology (57).

## Interactions between the complement system and enteric pathogens

Although numerous studies have examined the interplay between the complement system and enteric pathogens, much of the existing research has been conducted under in vitro conditions or with a focus on systemic infection. Local interactions between the complement system and pathogens in the gut remain underexplored. Here, we summarize studies that examine these local interactions and explore how enteric pathogens defend themselves against the complement system.

*Listeria monocytogenes* is a foodborne pathogen that breaches the intestinal epithelial barrier to invade the LP and subsequently disseminates through the lymphatic and blood systems. *L*. *monocytogenes* is a facultatively intracellular organism that can survive within both phagocytic and nonphagocytic cells (58). C3 is known to bind *L*. *monocytogenes* via the alternative pathway in human and mouse serum (59–61). Although its intracellular lifestyle protects the pathogen from extracellular complement, the host exploits C3 opsonization to restrict bacterial replication through autophagy. Within IECs, C3 directly interacts with ATG16L1, promoting autophagic clearance of the pathogen (62). Other intracellular pathogens, such as *Shigella flexneri* and *Salmonella enterica* serovar Typhimurium, evade this defense mechanism by secreting outer membrane proteases (IcsP and PgtE, respectively), which cleave surface-bound C3 to avoid autophagy. Notably, *Salmonella* PgtE also degrades human factor B and factor H, further suppressing alternative pathway activation (63). Adherent-invasive *E*. *coli* (AIEC) utilizes its LPS O-antigen, synthesized by the *wzy* gene, to shield itself from complement attack (64). Interestingly, colonization by the *wzy* mutant (which lacks LPS) alleviates DSS-induced colitis in a C3-dependent manner, suggesting context-dependent roles for complement in inflammation and microbial control.

*C. rodentium* is widely used as a model for human enteropathogenic *E*. *coli* infections (65). To establish infection, adherent *C*. *rodentium* spends several days adapting to the host environment (66), providing ample opportunity for local complement to opsonize the pathogen. Properdin (factor P) enhances host resistance to *C*. *rodentium* by amplifying complement activation and stimulating colonic epithelial IL-6 production, which reduces bacterial burden and inflammation (67). In 3- to 4-week-old mice, C3-deficient animals exhibit reduced survival following infection (16, 68). In adult mice, C3 was found to bind *C*. *rodentium* at the surface of colonic epithelium and in feces, and C3-deficient mice showed significant weight loss compared with WT controls (16). Interestingly, mice depleted of systemic C3 via cobra venom factor remained protected against *C*. *rodentium* infection, emphasizing the importance of local C3 (16). C3 deposition was detected in WT, C1q-deficient, and mannose-binding lectin–deficient (MBL-deficient) mice, suggesting that the alternative pathway likely mediates complement activation and deposition during *C*. *rodentium* infection (16).

It remains challenging to distinguish between local and systemic complement contributions during gut infection, especially under inflammatory conditions. Systemic complement influx can limit pathogen dissemination, as demonstrated by IL-22–driven C3 production during *Clostridioides difficile* infection, which targets translocating pathobionts in extraintestinal organs and reduces mortality (69). Similarly, systemic MBL restricts *Candida albicans* dissemination from the gut during experimental colitis (70).

Enteric parasites have also evolved mechanisms to circumvent complement-mediated defense in the gut. MBL deficiency has been associated with increased cryptosporidiosis in immunocompromised individuals and young children (71, 72). *Cryptosporidium parvum* activates both the classical and lectin complement pathways in vitro, with C1q and MBL contributing to host defense in gastrointestinal infection models (73). Similarly, MBL, H-ficolin, and L-ficolin bind *Giardia intestinalis*, a protozoan responsible for diarrheal disease and activate the complement lectin pathway to kill the parasite in human serum (74). However, in mice, MBL deficiency impairs Giardia clearance via a C3aR-dependent mechanism. This mechanism decreases mast cell recruitment rather than causing direct lysis (75).

The local complement pathway also plays an important role in enteric viral infections. Certain echovirus serotypes (6, 7, 11, 12, 20, and 21) and enterovirus 70 exploit the complement regulator CD55 as a receptor for attachment and infection (76, 77). Likewise, coxsackievirus serotypes B1, B3, B5, and A21 bind CD55 for cellular attachment, although this interaction alone does not mediate productive infection (78–80). Another CRP, CD46, also serves as a receptor for various viruses (81). These include measles virus (82, 83), which is capable of establishing persistent infection in the intestine (84); adenovirus serotypes 3, 11, 14, 16, 21, 35, 37, and 50 (85–88); and bovine viral diarrhea virus (89). Human astrovirus, a major cause of gastroenteritis, binds C1q and MBL via its coat protein, thereby disrupting the activation of the classical and lectin complement pathways (90, 91). Further studies have shown that the coat protein binds C1q at both the globular head region and the collagen-like region, which interacts with the serine protease tetramer (C1s-C1r-C1r-C1s), disrupting the integrity of the C1 complex. However, as most complement-virus interactions have been characterized in vitro, their biological relevance during natural infections requires further validation.

These findings underscore the complex relationship between the complement system and pathogens in the gut, highlighting both the protective and evasion mechanisms employed by various microbial agents. Further studies examining local complement activity in vivo are essential for a comprehensive understanding of this dynamic interaction.

## Gut complement, microbiota, and chronic inflammatory diseases

The complex role of the complement system in the development and progression of chronic inflammatory diseases of the gut, such as inflammatory bowel disease (IBD), has long been recognized. This complexity stems from complement’s capacity to both maintain and disrupt intestinal homeostasis. On one hand, complement supports tissue repair and homeostasis by clearing pathogens and damaged cells. On the other hand, excessive or dysregulated complement activation can trigger inflammation and drive tissue injury.

Substantial evidence points to both systemic and local complement dysregulation as a contributor in IBD. Patients with IBD exhibit elevated C3 synthesis rates compared with individuals acting as healthy controls, and patients with CD exhibit higher C3 production and serum levels than patients with UC (92). Likewise, C1q synthesis and serum concentrations are increased in individuals with CD relative to those in individuals with UC and healthy individuals (93), and elevated circulating levels of C3, C4, and factor B have been reported across IBD cohorts (94, 95). Direct detection of jejunal secretions in patients with CD further revealed increased C3 and C4 but not FB. Without concurrent albumin leakage, this finding suggests that localized complement synthesis contributes to these elevated levels (19).

Additionally, deposition of IgG1, C3b, and the MAC on the intestinal epithelium in patients with UC indicates classical pathway involvement (96). In contrast, C3b deposition in CD occurs without IgG, C1q, or C4c, pointing toward alternative pathway activation (97)

IECs are known to be an important source of C3 in IBD (15, 98). Recently, single-cell spatial transcriptomics combined with multiplexed error-robust fluorescence in situ hybridization (MERFISH) identified fibroblast subsets with high C3 expression in both healthy and inflamed tissues in murine colitis models (99). Similar patterns have been observed in patients with UC, suggesting that fibroblasts also contribute to C3 production under inflammatory conditions.

Our recent work suggests that the microbiota can regulate C3 levels in the gut (16). Given that IBD is associated with reduced microbial diversity and a shift from antiinflammatory species (e.g., *Faecalibacterium prausnitzii*, *Roseburia* spp.) to proinflammatory pathobionts (e.g., *E. coli*, *Ruminococcus gnavus*) (100, 101), it is plausible that microbial dysbiosis modulates C3 levels and activation in the gut, thereby exacerbating mucosal inflammation.

Although associations between microbiota composition and IBD pathogenesis are increasingly recognized, the underlying causal relationships and the potential involvement of the complement system are not yet fully elucidated.

## Gut complement and microbiota in CRC

CRC is the third most commonly diagnosed cancer worldwide and the second leading cause of cancer-related mortality (102). Emerging evidence implicates the complement system as a key player in CRC progression. Elevated expression of complement components and regulators, including C2, C3, C5, FB, FI, FHR1 (factor H–related protein 1), CR4 (ITGAX), C4BPB, CD46, CD55, and CPN1, has been reported in colon adenocarcinoma tissues (103), with notable activation of the lectin pathway in patients with CRC (104). However, complement signaling appears to exert context-dependent effects during CRC pathogenesis.

Serum levels of the anaphylatoxin C3a are increased in patients with CRC and decline following tumor resection, suggesting its potential utility as a biomarker (105–107). Paradoxically, in other cases C3aR expression has been observed to be epigenetically silenced in CRC via promoter hypermethylation, and loss of C3aR function promotes tumor development by enabling microbiota-driven infiltration of proinflammatory immune cells into colorectal tissues (108). Similarly, elevated plasma levels of C5a have been observed in patients with CRC (109), and overexpression of its receptor, C5aR1, enhances CRC cell proliferation, migration, and invasion in vitro (110). Tumor-associated macrophages further amplify C5a production through platelet-mediated activation of the JNK/STAT1 pathway, promoting tumor growth (111). C5a/C5aR1 signaling also exacerbates CRC progression by recruiting myeloid-derived suppressor cells that suppress CD8^+^ T cell activity in preclinical models (112) and by stabilizing β-catenin in epithelial cells via altered ubiquitination (113). Additionally, this axis promotes liver metastasis through PI3K/AKT-driven MCP-1–mediated recruitment of bone marrow–derived macrophages, neutrophils, and DCs (114). Collectively, while C3a/C3aR signaling exhibits dual, context-dependent roles, C5a/C5aR1 signaling consistently drives tumorigenesis and immunosuppression in CRC (115).

The microbiota-dependent production of C3 by intestinal cells and the minimal presence of C5 under homeostatic conditions in the gut (16) warrants further investigation to delineate the origins of complement components in CRC. Clarifying these pathways will be critical for identifying diagnostic biomarkers and therapeutic targets for CRC. The gut microbiota contributes to CRC through diverse mechanisms, including induction of genotoxic stress, activation of oncogenic signaling, promotion of chronic inflammation, immune evasion, and metabolic reprogramming (116). Meta-analyses of fecal sequencing data from patients with CRC have revealed enrichment of species such as *Fusobacterium nucleatum*, *Solobacterium moorei*, *Porphyromonas asaccharolytica*, *Parvimonas micra*, *Peptostreptococcus stomatis*, *P*. *anaerobius*, and *Prevotella intermedia* (117, 118). These findings support a model in which tumor-associated microbiota drive local complement expression, shaping the immune microenvironment to favor malignant progression.

Although the MAC can directly lyse tumor cells by disrupting membrane integrity (119), CRC cells evade complement-mediated cytotoxicity by upregulating CRPs such as CD55, CD46, and CD59 via STAT3/STAT6/p38 MAPK signaling (44, 107, 120, 121). Silencing these CRPs induces apoptosis in vitro and inhibits tumor growth in vivo (121), while anti-CD55 antibodies restore complement activation and suppress CRC proliferation and invasion (122). High CRP expression correlates with poor prognosis in patients with CRC (123, 103), highlighting their potential as therapeutic targets. In addition, factor I has been shown to promote CRC growth by enhancing glycolytic flux through the Wnt/β-catenin/c-Myc axis (124), and the accumulation of factor H and its splice variant FHL-1 in liver metastases may further inhibit MAC formation and promote tumor survival (125).

## Future perspectives

Collectively, our emerging understanding of gut complement biology positions this ancient immune pathway as a critical mediator of intestinal homeostasis and disease. Its dual capacity to shape microbiota-host interactions and regulate barrier function underscores the complement system’s unique role in maintaining the health of the gut ecosystem. However, when dysregulated, complement can become a potent driver of chronic inflammatory diseases, metabolic disorders, and malignancies. As research continues to unravel the microbial and host-derived signals that govern complement production and activation in the gut, new therapeutic strategies are on the horizon. Precision targeting of complement pathways — whether through modulation of microbial communities, restoration of complement regulation, or inhibition of pathogenic activation — holds promise for a new class of therapeutics aimed at reestablishing mucosal balance. Future studies delineating the spatial, cellular, and molecular determinants of gut complement activity will be pivotal in translating these insights into clinical interventions that improve outcomes across a spectrum of gastrointestinal and systemic diseases.

## Figures and Tables

**Table 3:**
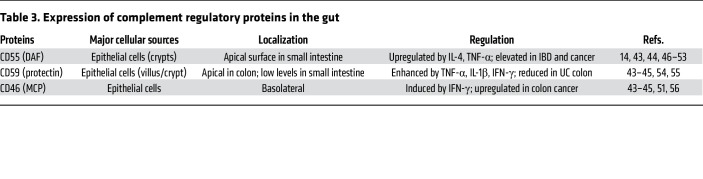
Expression of complement regulatory proteins in the gut

**Table 2:**
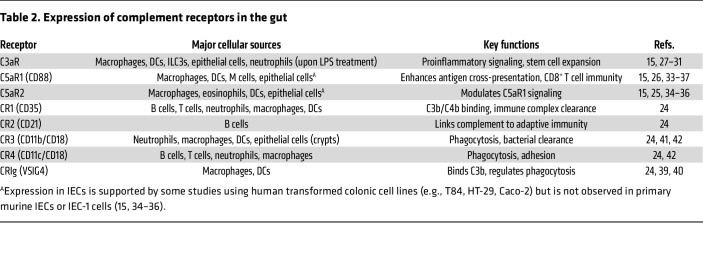
Expression of complement receptors in the gut

**Table 1:**
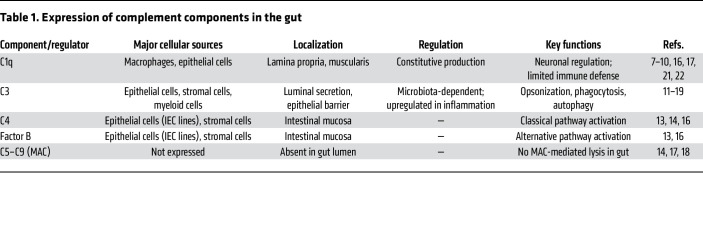
Expression of complement components in the gut

## Version 1 10/01/2025

Electronic publication
